# Morphological characteristics of acquired corneal sub-epithelium hypertrophy: a case series

**DOI:** 10.3389/fmed.2026.1829840

**Published:** 2026-06-17

**Authors:** Qiaoyu Li, Yunfan Zhang, Haimiao Lin, Zhaoxiang Lu, Wenyu Wu, Yun Feng

**Affiliations:** Department of Ophthalmology, Peking University First Hospital, Beijing, China

**Keywords:** acquired corneal sub-epithelium hypertrophy, corneal transplantation, histopathology, immunofluorescence, optical coherence tomography

## Abstract

**Introduction:**

Acquired Corneal Subepithelial Hypertrophy (ACSH) is a rare corneal opacity secondary to ocular surgery or trauma. It is characterized by subepithelial fibrosis, a marked male predominance, and a high risk of misdiagnosis due to non-specific slit-lamp findings. This study aimed to characterize the *in vivo* morphological features of ACSH using anterior segment optical coherence tomography (AS-OCT) and clarify its histopathological composition.

**Methods:**

This retrospective single-center case series enrolled 11 patients (12 eyes) with ACSH who underwent surgical intervention at our institution between 2018 and 2022. AS-OCT imaging was performed to evaluate subepithelial fibrosis features. All specimens underwent histopathological evaluation with hematoxylin and eosin (HE), periodic acid-Schiff (PAS), and Masson trichrome staining. Immunofluorescence analysis was conducted on five specimens to assess cytokeratins (CK3, CK14), nucleoprotein (P40-ΔNp63), cytoskeletal proteins (alpha-smooth muscle actin, vimentin), and extracellular matrix components (Collagen I, Collagen IV). Primary outcomes included AS-OCT morphological characteristics from baseline to final follow-up and corresponding histopathological findings.

**Results:**

Most patients were male, and the primary presenting symptom was blurred vision. Superficial keratectomy restored corneal clarity without recurrence during follow-up. ACSH was categorized into three subtypes: paracentral patchy opacification (PPO, 41.7%), peripheral sectorial nodules (PSN, 41.7%), and central diffuse mass (CDM, 16.7%). AS-OCT revealed hyperreflective lamellar deposits between the epithelium and stroma. The maximum thickness of fibrosis was strongly correlated with corneal surface thickness (*r* = 0.96; *p* < 0.0001). Histopathological findings suggested that corneal injury from surgery or trauma may drive subepithelial fibrosis via abnormal tissue repair and disorganized extracellular matrix deposition.

**Discussion:**

AS-OCT enables reliable identification of ACSH and differentiation from corneal leucoma and haze. Histopathological observations provide insights into potential pathogenic mechanisms. These findings may inform clinical strategies to prevent ACSH, such as using topical medications or contact lenses to enhance epithelial healing after anterior segment surgery or injury.

## 1 Introduction

Corneal opacity is the fifth leading cause of blindness and visual impairment worldwide. It represents a growing threat to vision, resulting from multiple etiologies, including corneal injury (1). Accurate identification of the affected corneal layers is essential, as management strategies vary substantially across different types of opacity. Most existing research has focused on subepithelial stromal scarring of varying severity, which can present as transient haze or permanent fibrotic scar tissue (2–4). Common treatments include topical mitomycin C and corneal transplantation, both approaches are associated with notable limitations (4, 5).

In contrast, subepithelial fibrosis above the stromal layer remains understudied. One example of this condition is a recently defined entity: acquired corneal subepithelial hypertrophy (ACSH). To date, only 15 ACSH patients (20 affected eyes) have been reported globally. The condition shows a strong male predominance. ACSH typically occurs after corneal surgery and is usually unilateral, affecting the operated eye. The morphological features of affected eyes are variable (6, 7). The pathogenesis and phenotypic spectrum of ACSH remain poorly characterized. Corneal thickness generally increases with the degree of opacification. In most cases, simple excision of the abnormal epithelium and subepithelial tissue restores corneal clarity without recurrence, often avoiding the need for corneal transplantation (6, 7). This form of subepithelial fibrosis may be misdiagnosed as corneal haze or leucoma. Slit-lamp examination alone is insufficient to determine the precise depth and layer involvement. Postoperative histopathological analyses have identified a superficial, non-inflammatory fibrotic layer between the epithelium and Bowman’s layer, with an underlying intact stroma. Therefore, a reliable and noninvasive diagnostic modality is important to prevent unnecessary corneal transplantation due to misdiagnosis.

Anterior segment optical coherence tomography (AS-OCT) has become a valuable preoperative tool for noninvasive evaluation of the anterior corneal layers. It enables detailed visualization and characterization of ACSH-related opacities and provides cross-sectional imaging of the anterior segment in affected patients (8). However, the diagnostic value of AS-OCT for ACSH has not been systematically explored. Furthermore, a better understanding of the mechanisms underlying ACSH may optimize clinical care. Our study was designed to evaluate the clinical and morphological features of different ACSH phenotypes using AS-OCT and to explore potential pathogenic mechanisms through histopathological examination in 11 patients with typical disease.

## 2 Methods

### 2.1 Patients

This retrospective single-center case series analyzed patients clinically diagnosed with atypical corneal stromal haze at Peking University Third Hospital between 2018 and 2022. The study was approved by the Institutional Review Board of Peking University Third Hospital Medical Science Research Ethics Committee and was conducted in accordance with the Declaration of Helsinki. A total of 11 patients, accounting for 12 affected eyes, were included. Eligible patients had a clinical diagnosis consistent with atypical corneal stromal haze, complete slit-lamp or other clinical imaging records, and anterior segment optical coherence tomography images. For a subset of cases, corneal tissue obtained through superficial keratectomy was available for histopathological examination. Typical cases were characterized by unilateral or bilateral subepithelial fibrosis located between the epithelium and Bowman’s layer with a clearly defined demarcation line, whereas stromal scarring was not considered. Patients without biopsy confirmation were excluded from the study.

Cases were further excluded if clinical or imaging data were incomplete, if anterior segment optical coherence tomography images were of insufficient quality for reliable analysis, if other ocular conditions known to cause corneal haze were present, or if atypical corneal stromal haze could not be clearly distinguished from other corneal pathologies. All included cases were reviewed, and slit-lamp and optical coherence tomography images were independently evaluated by two experienced cornea specialists. Histopathological findings, when available, were used to corroborate clinical and imaging assessments. Recurrence was defined as the reappearance of abnormal fibrous deposits between the epithelium and stroma. Applying these predefined criteria ensured the reliability and consistency of the dataset.

### 2.2 Data collection

All eyes underwent comprehensive ophthalmic examinations. Digital slit-lamp photographs of the cornea were obtained using a slit-lamp biomicroscope (EyeCap v7 version 7.5.28). AS-OCT scanning was performed before treatment and during follow-up using an RTVue XR Avangi device (Optovue Inc., Fremont, CA, USA) with corneal line mode via an anterior segment optical adapter lens. Four corneal images (superior, inferior, nasal, and temporal) were acquired for each patient under fixation, with an axial scanning resolution of 5 μm.

**Figure 1:**
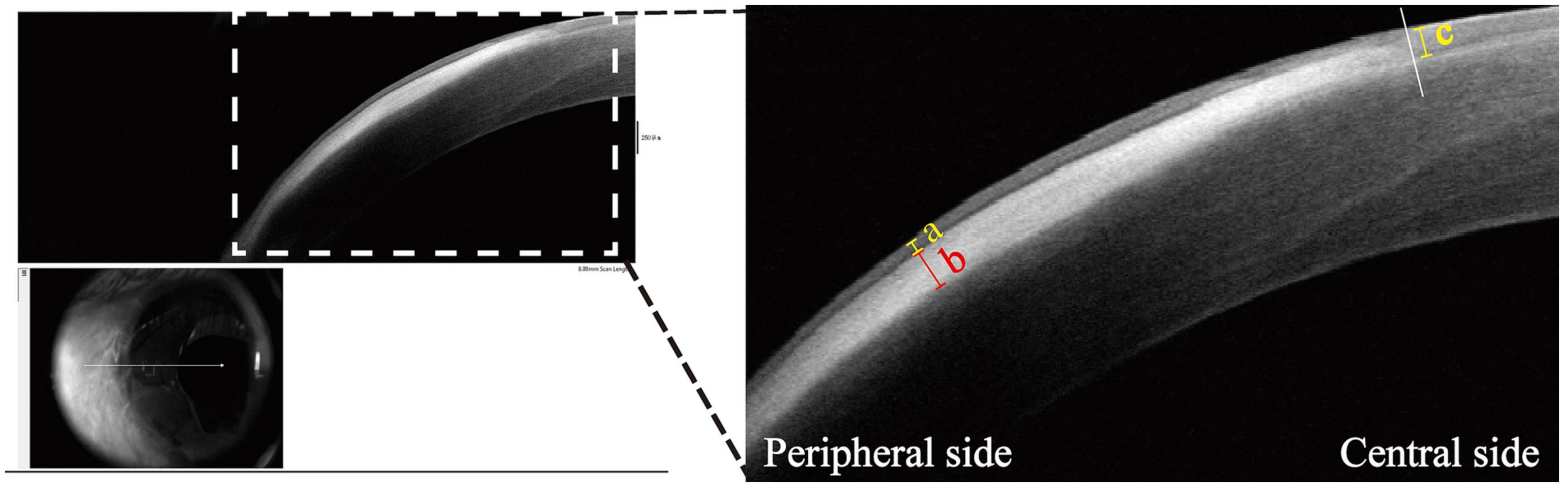
Schematic diagram illustrating the thickness measurement of ACSH lesions. a: Thickness of the overlying epithelium above fibrosis (yellow lines); b: Thickness of the fibrotic layer (red lines); c: Thickness of normal corneal epithelium (yellow lines).

The AS-OCT image showing the thickest subepithelial fibrosis was selected for each patient and used for thickness measurements (Figure 1). The thickness of the normal epithelium was measured at the edge of the fibrosis closest to the corneal center. Epithelial thickness was measured between the corneal surface and the superficial margin of the fibrosis. Fibrosis thickness was measured between the superficial and posterior margins of the fibrosis. Corneal surface thickness was calculated as the sum of epithelial thickness and fibrosis thickness.

### 2.3 Sample collection

Surgical excision was performed to relieve patient symptoms and completely resect subepithelial fibrosis at the interface between the lesion and healthy stroma. All procedures were performed by a single surgeon (F. Y.) under topical anesthesia. Antimetabolites were not used in any case. All 11 patients (12 eyes) underwent histopathological examination. Eight excised tissue specimens were immediately embedded in O. C. T. compound (TissueTek; Sakura Finetek Co., Ltd., Tokyo, Japan) and snap-frozen in liquid nitrogen. Four specimens were fixed in 10% neutral-buffered formalin, dehydrated, and embedded in paraffin.

### 2.4 Histopathologic stains

All specimens were sectioned at 5 μm and stained with Hematoxylin and Eosin (HE), Periodic Acid-Schiff (PAS), and Masson trichrome stain. In 5 surgical samples from 5 patients, sufficient tissue was available for immunohistochemical staining.

Briefly, cryosections (5 μm thick) were air-dried, fixed with acetone for 8 min, and immersed in blocking solution (10% goat serum in 0.01 M PBS) for 1 h. Primary antibodies included CK3 (Clone AE5, Abcam, 1:200), CK14 (Clone LL002, Abcam, 1:200), P40-ΔNp63 (Clone EPR17863-47, Abcam, 1:100), alpha-smooth muscle actin (Clone 1A4, Abcam, 1:200), Vimentin (Clone EPR3776, Abcam, 1:200), Collagen I (Clone EPR7785 138,492, Abcam, 1:200), and Collagen IV (Clone EP1254, Abcam, 1:200). Sections were incubated with primary antibodies overnight at 4 °C. Sections were washed three times with 0.01 M PBS and then incubated with fluorescent secondary antibody solutions (Alexa 488– or 594–labeled anti-mouse IgG or anti-rabbit IgG). After incubation at 37 °C for 1 h, sections were washed with 0.01 M PBS, and nuclei were counterstained with DAPI. Unless otherwise stated, all incubations were performed at room temperature. After a final wash with PBS, sections were mounted. All images were captured using a digital camera (LSM510; Carl Zeiss Inc.). Negative control experiments were performed for each antibody under identical immunostaining conditions and showed no positive staining.

### 2.5 Statistical analysis

Statistical analysis was performed using SPSS software version 26.0 (IBM Corp., Armonk, NY, USA). Continuous variables were expressed as mean ± standard deviation (SD). Correlations between thickness measurements were evaluated using Pearson correlation coefficient. A *p*-value < 0.05 was considered statistically significant.

## 3 Results

### 3.1 Demographic and clinical characteristics

**Figure 2:**
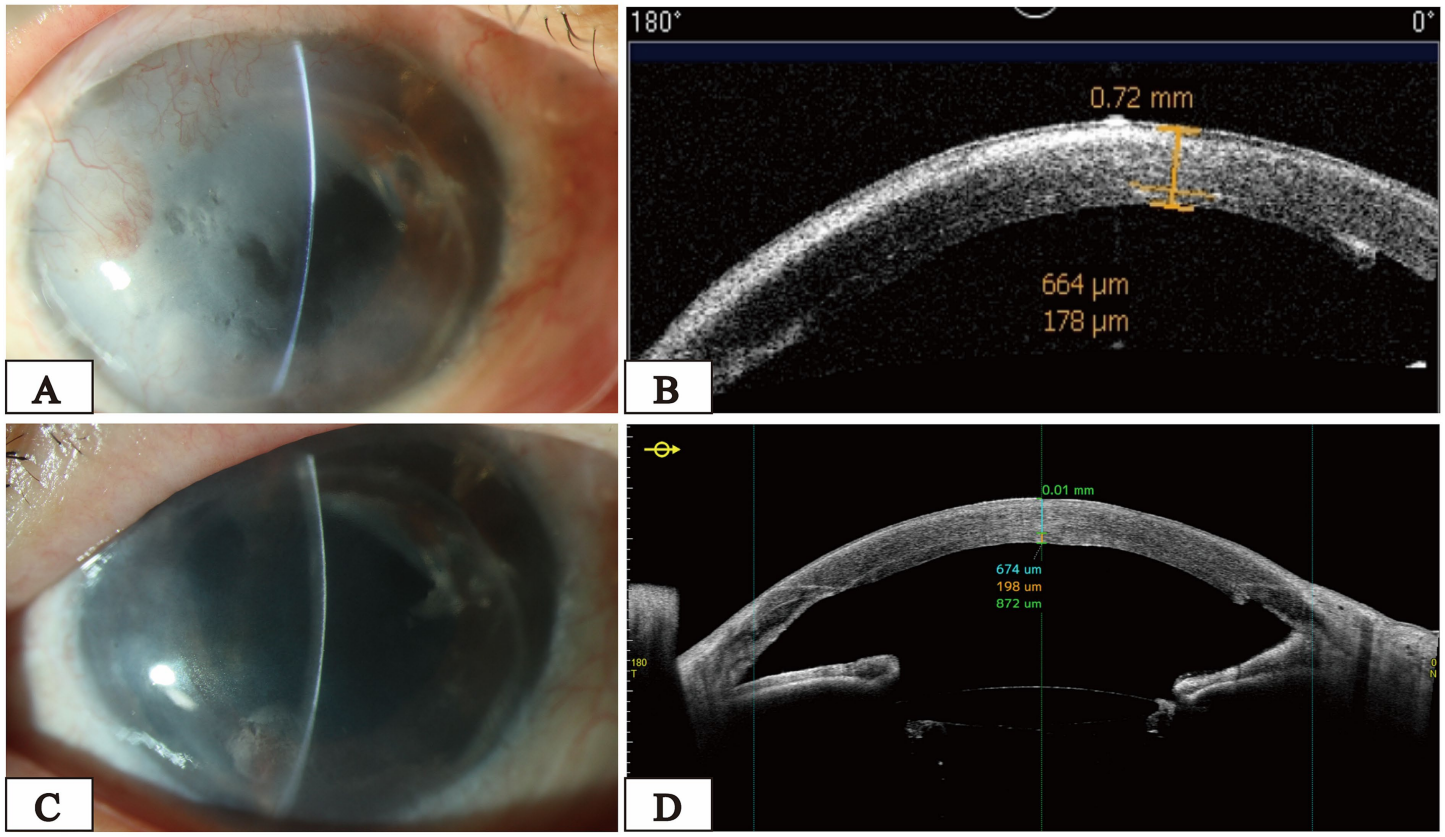
Slit-lamp and AS-OCT images of patient 11 with PPO before surgery **(A,B)** and 917 days after superficial keratectomy **(C,D)**, showing no postoperative recurrence.

The demographic and clinical characteristics of the 11 patients are summarized in Table 1. Only one patient (9%) presented with bilateral thickened opacification, and 7 of 12 affected eyes were right eyes. The median age was 43 years (range, 28–85 years). Nine patients were male, and two were female. The median follow-up duration was 39.5 days (range, 20–917 days). All patients had a history of ocular surgery or trauma. Seven of 12 eyes (58.3%) had undergone corneal transplantation, including 3 eyes (42.9%) with penetrating keratoplasty (PKP), 3 eyes (42.9%) with endothelial keratoplasty (EK), 1 eye (14.3%) with repeated EK, and 1 eye (14.3%) with deep lamellar keratoplasty (DLK). The remaining 5 eyes (41.7%) had a history of trauma or anterior segment surgery, including cataract surgery and glaucoma surgery. No signs of chronic ocular surface inflammation or comorbidities were observed. The most common presenting symptom was decreased visual acuity, noted in 7 patients (63.6%). Opacified epithelium and subepithelial tissue were surgically removed in all eyes. No antimetabolites were used in any case. Superficial keratectomy restored corneal clarity in the treated cases during the available follow-up period. Symptomatic relief was achieved in all eyes. No recurrence was observed within the limited follow-up period; however, only one patient had long-term follow-up, and the median follow-up was short. Therefore, no definitive conclusion regarding recurrence risk can be drawn. Images from patient 11, who had the longest follow-up period of 917 days, are presented in Figure 2. Recurrence could not be definitively assessed due to the small sample size and limited follow-up in other patients.

**Table 1:** Demographic characteristics of 11 patients with typical acquired corneal sub-epithelium hypertrophic (ACSH).

ID	Sex	Age (year)	EYE	Symptoms	History of corneal transplantation	The presenting day after transplantation	Trauma	History of anterior segment surgery	Operation	Follow-up days	Recurrence
1	M	32	OD	Loss of vision	N	506	Y	Y	SK	20	N
2	M	62	OS	Loss of vision	N	——	Y	Y	EK + SK	38	N
3	M	85	OU	Blurred vision	N	——	Y	Y	EK + SK	41	N
4	M	28	OS	Loss of vision	N	3,017	Y	N	SK	N	N
5	M	65	OS	Blurred vision	PKP	885	0	Y	SK	122	N
6	M	43	OD	Blurred vision	EK	434	Y	Y	EK + SK	N	N
7	M	50	OD	Asymptomatic	EK	154	0	Y	SK	20	N
8	M	39	OS	Irritation	DALK	473	0	N	SK	N	N
9	F	42	OD	Blurred vision	PKP	483	0	N	EK + SK	N	N
10	M	39	OD	Blurred vision	PKP	922	Y	Y	SK		N
11	F	62	OD	Blurred vision	2EK	419	0	Y	SK	917	N

M, Male; F, Female; OD, Right eye; OS, Left eye; OU, Right and left eyes; CDM, Central diffuse mass; PPO, Paracentral patchy opacification; PSN, Peripheral sectorial nodules; Y, Yes; N, No; PKP, Penetrating keratoplasty; SK, Superficial keratectomy; EK, Endothelial keratoplasty.

### 3.2 Slit lamp microscope observation

**Figure 3:**
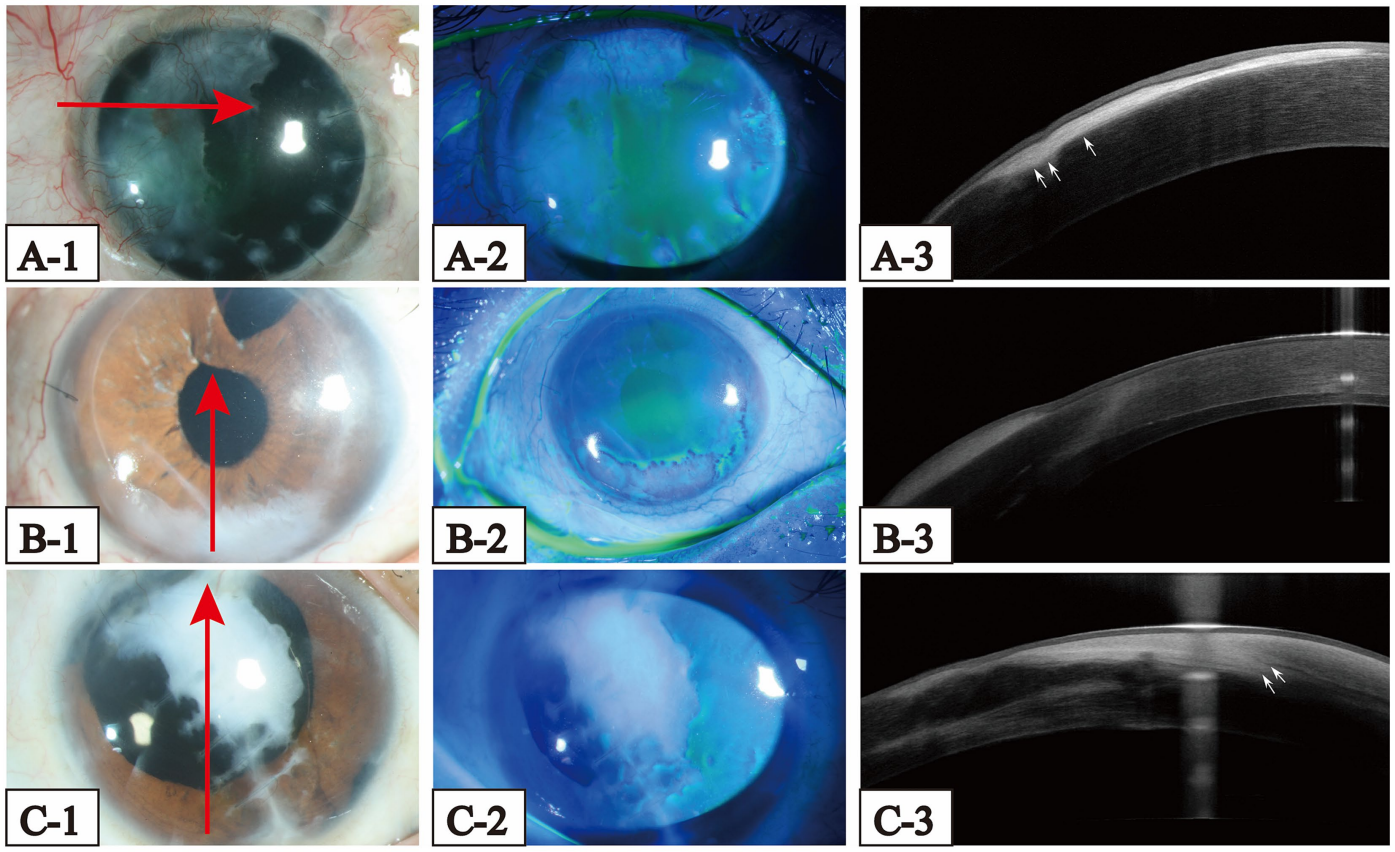
Slit-lamp diffuse photographs, fluorescein staining and AS-OCT images of three representative ACSH phenotypes. **(A)** PSN (patient 10); **(B)** PPO (patient 6); **(C)** CDM (patient 1). All cases showed negative fluorescein staining (A-2, B-2, C-2). Red arrows in A-1, B-1 and C-1 indicate the scanning direction of AS-OCT. AS-OCT confirmed focal hyperreflective superficial fibrotic deposits located between the epithelium and Bowman’s layer. Discontinuity of Bowman’s layer is marked by white arrows.

Representative slit-lamp photographs, fluorescein staining findings, and AS-OCT images of the three distinct phenotypic subtypes are presented in Figure 3. All lesions were associated with an intact corneal epithelium, and fluorescein staining results were negative. The lesions could be classified into three subtypes: paracentral patchy opacity (PPO), peripheral sectorial nodules (PSN), and central diffuse mass (CDM). Among the patients, 5 cases (41.7%) were PPO, 5 cases (41.7%) were PSN, and 2 cases (16.7%) were CDM.

PPO was characterized by a curved, patch-like shape with irregular margins, and obvious vascularization was observed at the root of the fibrous tissue. PSN presented as discontinuous crescent-shaped nodules, mostly located in the inferior corneal region, accompanied by slight corneal flattening. CDM manifested as a compact, diffuse mass involving the central corneal region, which was closely associated with visual axis involvement, resulting in a distinct decrease in visual acuity.

Patients with PPO and PSN were all secondary to corneal transplantation, while those with CDM were mostly secondary to other ocular surgeries or trauma. The clock-hour distribution of subepithelial fibrosis revealed a predominance of the inferior region, with detailed data summarized in Table 2.

**Table 2:** The details of different phenotypes in slit-lamp and OCT.

ID	Eye	Phenotype	Location (Clock hours)	Interruption of Bowman’s layer	Epithelial thickness above the fibrosis(μm)	Maximum thickness of fibrosis(μm)	Thickness of corneal surface(μm)	Thickness of normal epithelium(μm)
1	OD	CDM		Y	68.7	340.0	408.7	70.7
2	OS	CDM		N	66.7	176.0	242.7	49.7
3	OS	CDM		N	66.3	252.7	319.0	92.3
3	OD	CDM		N	68.7	258.0	326.7	89.7
4	OS	CDM		N	21.0	311.7	332.7	56.3
5	OS	PSN	3–5, 10–11	Y	68.0	395.3	463.3	70.7
6	OD	PSN	5–7, 7–8	N	50.7	205.7	256.3	78.0
7	OD	PSN	2–4	N	29.3	103.7	133.0	59.3
8	OS	PSN	3–4, 4–5	Y	26.0	264.3	290.3	87.7
9	OD	PSN	5–7	Y	13.3	83.0	96.3	47.3
10	OD	PPO	3, 4–2	Y	66.7	221.0	287.7	74.0
11	OD	PPO	3, 4–5, 5–2	N	45.0	179.3	224.3	62.3

OD, Right eye; OS, Left eye; CDM, Central diffuse mass; PPO, Paracentral patchy opacification; PSN, Peripheral sectorial nodules; Y, Yes; N, No.

### 3.3 AS-OCT findings

**Figure 4:**
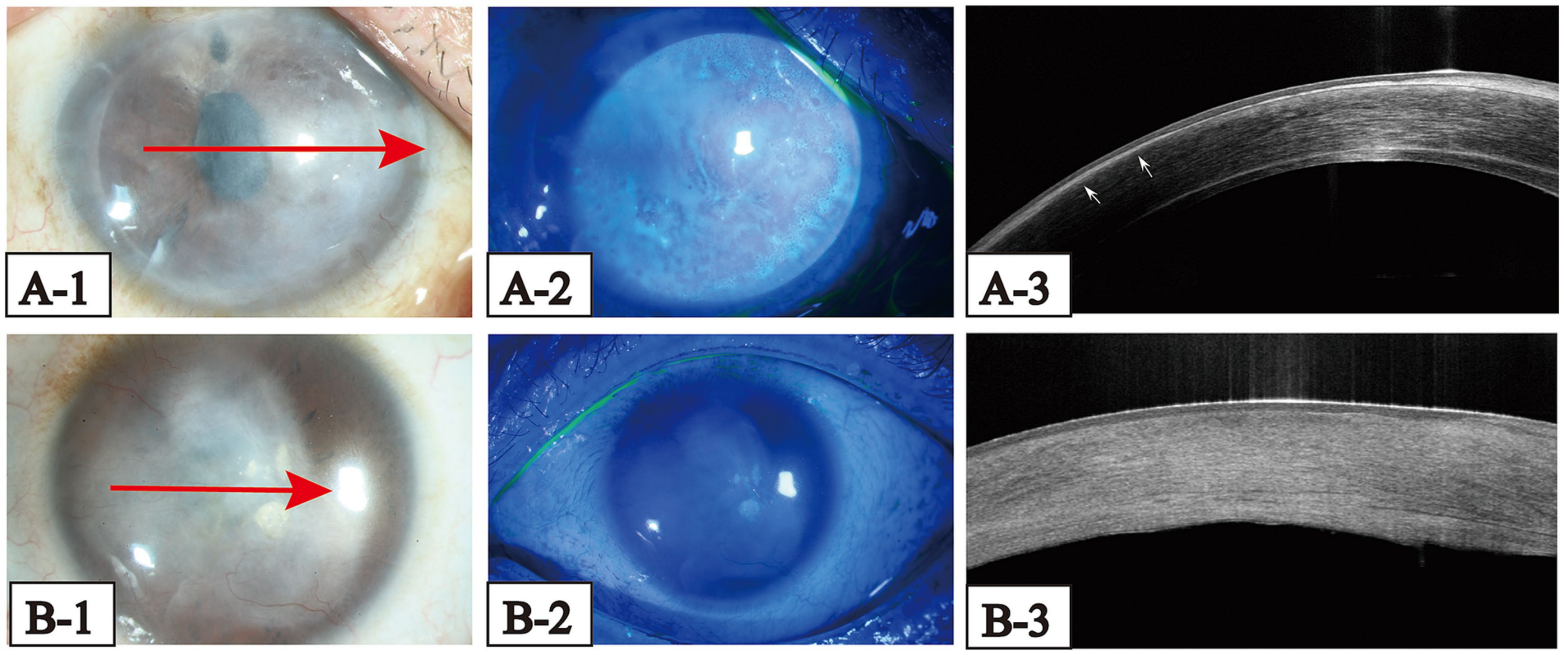
Comparative slit-lamp and AS-OCT findings between ACSH and stromal scarring. **(A)** ACSH case (patient 7); **(B)** Stromal scarring control case; 1: Slit-lamp diffuse image; 2: Fluorescein staining; 3: AS-OCT image. Prominent hyperreflective lamellar deposits in ACSH (white arrows) are located between the epithelium and stroma above Bowman’s layer, with well-defined upper and lower boundaries. In stromal scarring, no subepithelial fibrosis was observed; instead, heterogeneous hyperreflective changes involved the full-thickness stroma.

AS-OCT imaging of patients with ACSH revealed a “sandwich” superficial structure (Figures 3C, 4A). A prominent hyperreflective lamellar deposit was observed between the corneal epithelium and stroma. This deposit was located above Bowman’s layer, with clear upper and lower boundaries. Compared with the adjacent normal corneal surface, all cases showed elevation of the anterior corneal surface, reduced transparency, and regional thickening, which was consistent with significant epithelial thinning. The subepithelial fibrosis appeared as a distinct layer with homogeneous signal intensity and a smooth superficial margin. All lesions had a clear demarcation line from the superficial stroma. In 5 cases with a disrupted Bowman’s layer and an irregular posterior margin, sporadic infiltration into the deeper stroma was observed (Table 2). For the control case with known stromal scarring, slit-lamp examination showed opacity similar to that of ACSH. However, AS-OCT could easily distinguish the two conditions: the control case showed no subepithelial fibrosis, but rather heterogeneous and hyperreflective changes throughout the entire stromal thickness (Figure 4B).

**Figure 5:**
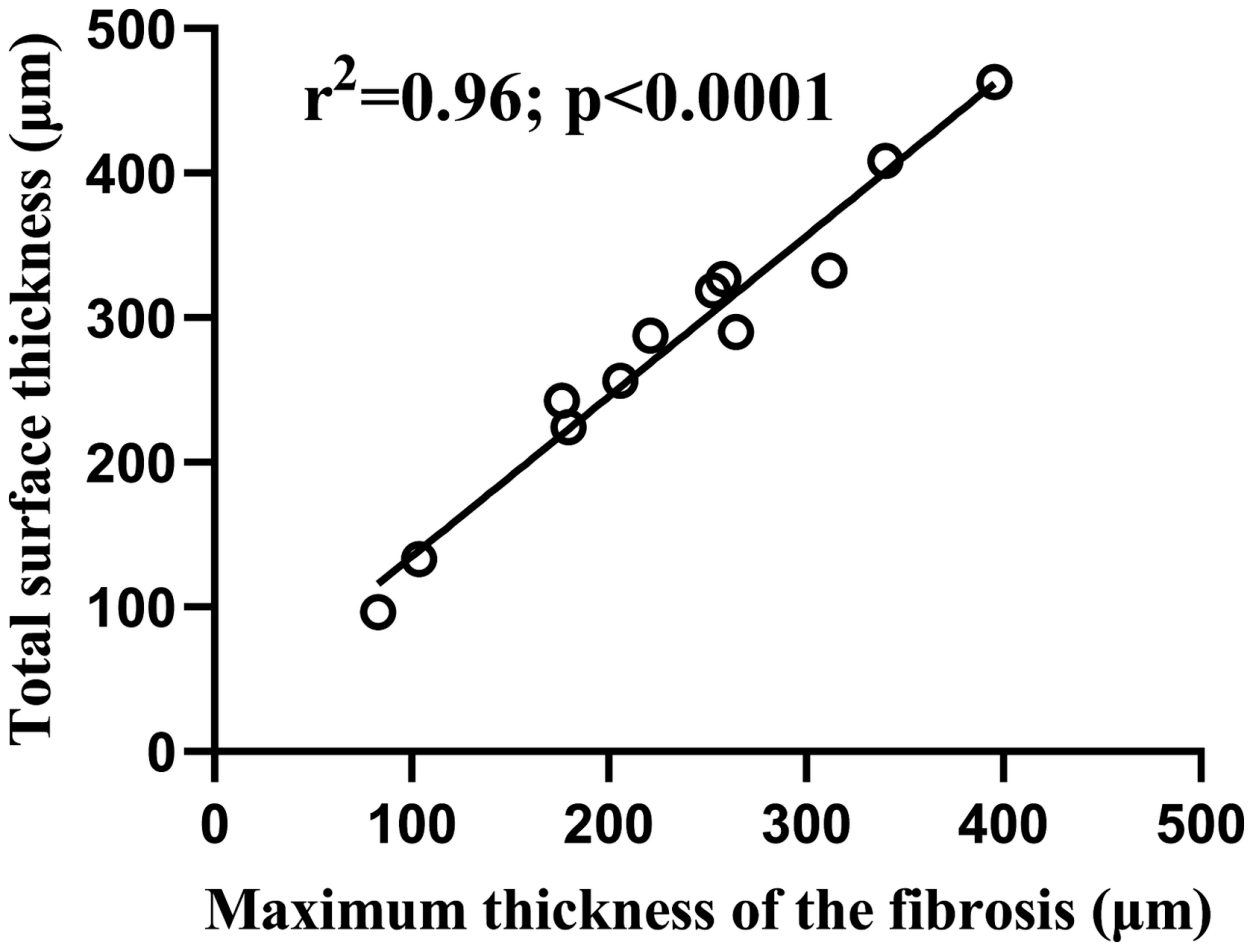
Scatter plot showing the correlation between total corneal surface thickness and maximum fibrotic thickness (r^2^ = 0.96; *p* < 0.0001).

The epithelial thickness above the fibrosis and the maximum thickness of the fibrosis varied among patients with ACSH (Table 2). The mean epithelial thickness above the fibrosis was 49 ± 21 μm (range: 13–69 μm). The mean maximum thickness of the fibrosis was 233 ± 92 μm (range: 83–395 μm). The mean thickness of the normal corneal epithelium was 70 ± 15 μm (range: 47–92 μm). A statistically significant positive correlation was found between the maximum thickness of the fibrosis and the thickness of the corneal surface (*r* = 0.96; *p* < 0.0001; Figure 5). Because of the retrospective nature of this study, standardized functional outcomes, including best-corrected visual acuity and serial corneal thickness measurements, were not available for all patients. Therefore, we were unable to perform a systematic quantitative analysis of functional improvement or structural change.

### 3.4 Histopathology

**Figure 6:**
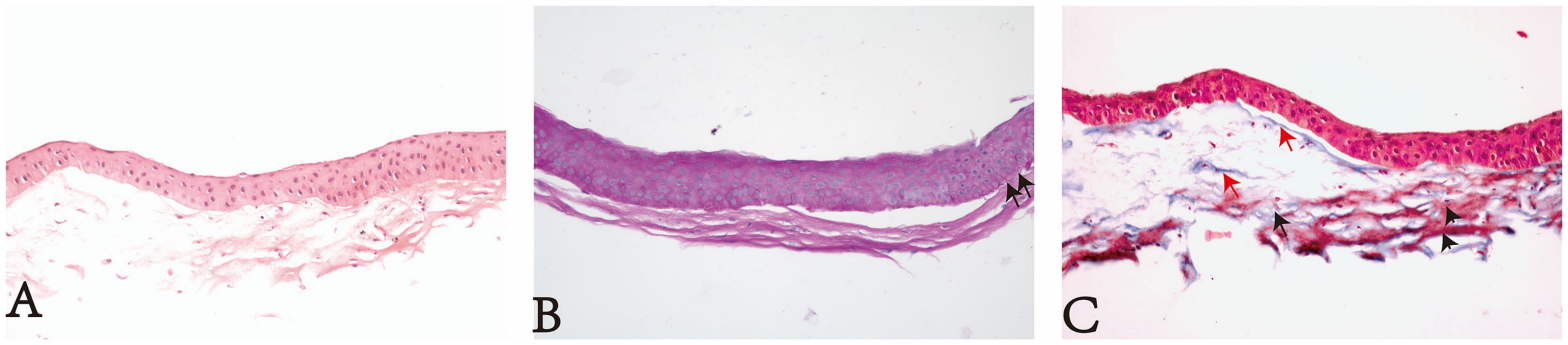
Light micrographs of routine histological staining. **(A)** HE staining; **(B)** PAS staining; **(C)** Masson trichrome staining. Original magnification: ×200. HE and PAS staining showed that the superficial stratified eosinophilic epithelium consisted of superficial squamous cells, wing cells and basal columnar cells, with a thin and discontinuous basement membrane (black arrow, **B**). The underlying hypocellular fibrotic tissue presented a continuous regular surface with eosinophilic fibers and scattered fibroblast-like cells. Masson trichrome staining revealed abundant blue-stained collagen fibers (red arrow, **C**) and sparse red-stained muscle fibers (black arrow, **C**).

All enrolled patients were confirmed to have ACSH histopathologically. The superficial eosinophilic epithelium exhibited a stratified architecture comparable to that of normal corneal tissue (Figure 6A). The epithelial basement membrane appeared thin and discontinuous (black arrows, Figure 6B). The underlying hypocellular fibrosis presented as continuous lamellar tissue without vascularization, composed of parallel-arranged collagen fibers (red arrow, Figure 6C) and sparse muscle fibers (black arrow, Figure 6C). Bowman’s layer was absent in most specimens, with remnants occasionally retained within the surgical bed. Immunofluorescence results are illustrated in Figure 5, 6.

**Figure 7:**
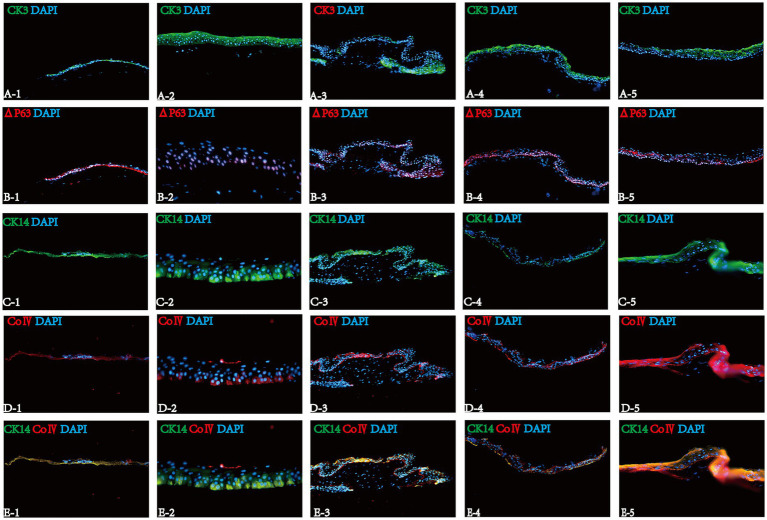
Expression distribution of cellular markers in the corneal epithelium. **(A)** CK3 / Nuclei; **(B)** ΔNp63 / Nuclei; **(C)** CK14 / Nuclei; **(D)** Collagen IV / Nuclei; **(E)** CK14 / Collagen IV / Nuclei. (1) CDM (patient 2); (2) CDM (patient 3); (3) PSN (patient 5); (4) PSN (patient 7); (5) PPO (patient 8). CK3 immunoreactivity gradually decreased from the superficial layer to the basal layer, while CK14 was strongly expressed in basal cells. ΔNp63 was localized in the nuclei of basal and wing cells. Collagen IV was detected in the cytoplasm of CK14-positive basal cells. Original magnification: (A 1–5, B–E 1, B–E 3–5): ×200; (B–E 2): ×400.

**Figure 8:**
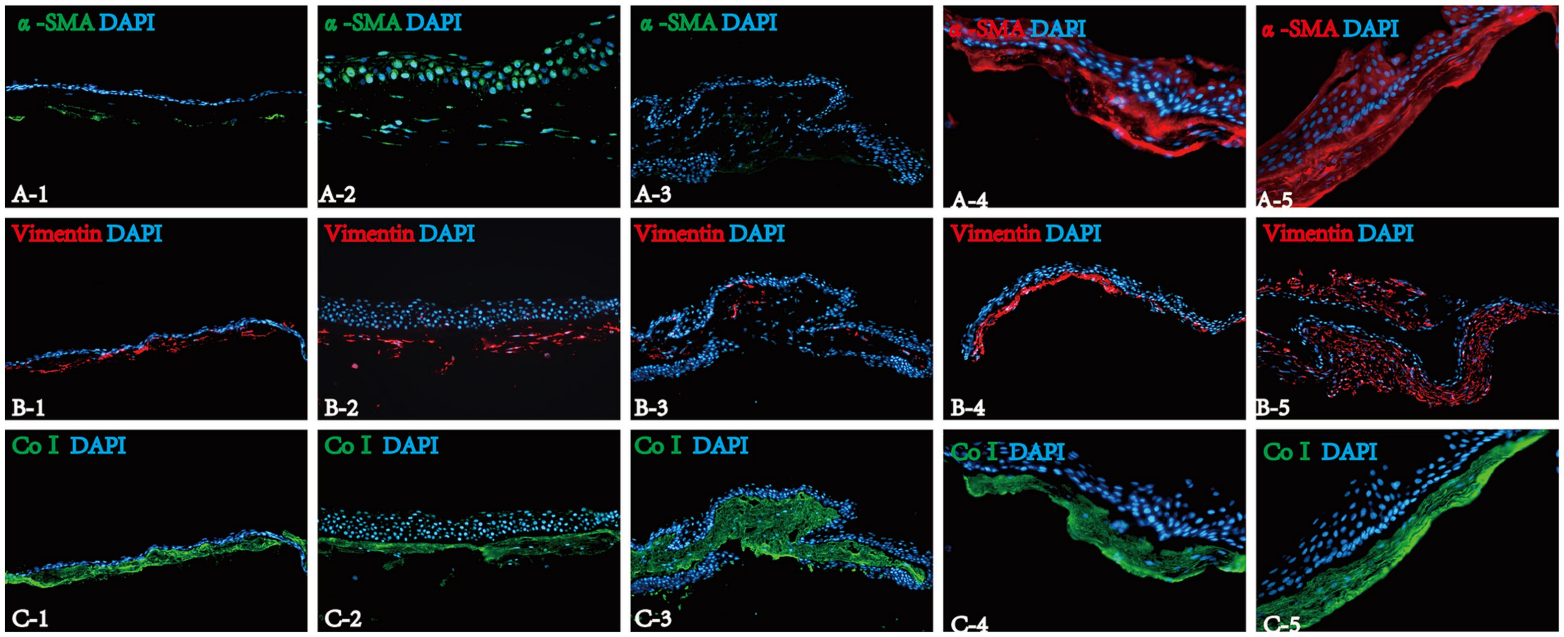
The distribution of markers in sub-epithelium fibrosis **(A)**. *α*-SMA/nuclei **(B)** Vimentin/nuclei; **(C)** Collagen I/nuclei (1) CDM (patient 2), (2) CDM (patient 3), (3) PSN (patient 5); (4) PSN (patient 7), (5) PPO (patient 8). The underlying fibrosis was diffusely immunoreactive to Collagen I. The fibroblast-like cells were immune-positive for vimentin, and myofibroblast-like cells characterized by α-SMA closer to the underlying stroma. Original magnification:(A-C 1, B-C 2, A-C 3, B 4–5): ×200, (A-2, A-C 4–5): ×400.

The epithelial cell population comprised three cell phenotypes consistent with normal corneal epithelium: CK14++/Np63++ basal cells, Np63+/CK3 + wing cells, and CK3 + superficial cells. Collagen IV was expressed in the cytoplasm of CK14-positive basal cells (Figure 7 E1–E5). Vimentin-positive fibroblast-like cells were distributed sporadically within the fibrotic tissue, while *α*-SMA-positive myofibroblast-like cells were predominantly detected in the deeper layers (Figure 8). These findings suggest that fibroblast activation may be involved in ACSH formation, although further mechanistic studies are needed.

## 4 Discussion

Acquired corneal subepithelial hypertrophy (ACSH) is a secondary corneal opacity that typically arises after penetrating keratoplasty, deep lamellar keratoplasty, keratectomy, or chronic ocular inflammation (6, 7). In this retrospective series of 11 patients involving 12 eyes, we observed a predominance of male patients across a wide age range, with non-specific presenting symptoms. Conventional slit-lamp biomicroscopy and corneal topography provide only macroscopic information about lesion morphology and corneal thickness (6, 7), which can lead to misdiagnosis as Salzmann nodular degeneration or post-traumatic/post-inflammatory stromal scarring. This limitation may partly explain why ACSH is underrecognized. Relying solely on slit-lamp examination risks underestimating the disease, and grouping all corneal opacities as a single entity is inappropriate. Management should be guided by subtype-specific features, underscoring the value of reliable preoperative imaging.

This study is the first to describe *in vivo* morphological features of ACSH using anterior segment optical coherence tomography (AS-OCT). Lesions appeared as homogeneous, hyperreflective, continuous fibrotic layers located between the epithelium and Bowman’s layer, with smooth surfaces and sharply demarcated boundaries, suggesting relatively simple tissue composition and organized structural arrangement. Histopathology confirmed that the fibrotic tissue was primarily composed of horizontally arranged type I collagen fibers with sparse muscle fibers, with minimal inflammatory infiltration (3, 5, 8). Quantitative AS-OCT measurements indicated that thickened lamellar lesions contributed to anterior corneal surface elevation, while epithelial thickness remained largely unaffected. Immunofluorescence analysis showed that basal epithelial cells retained self-renewal capacity, with CK3 expression gradually decreasing from the superficial to basal layers and ΔNp63 localized in the nuclei of wing and basal cells, consistent with the rapid epithelial regeneration observed after keratectomy. Simple epithelial debridement or superficial keratectomy restored corneal clarity effectively and was associated with a low recurrence rate, avoiding the need for penetrating keratoplasty.

**Figure 9:**
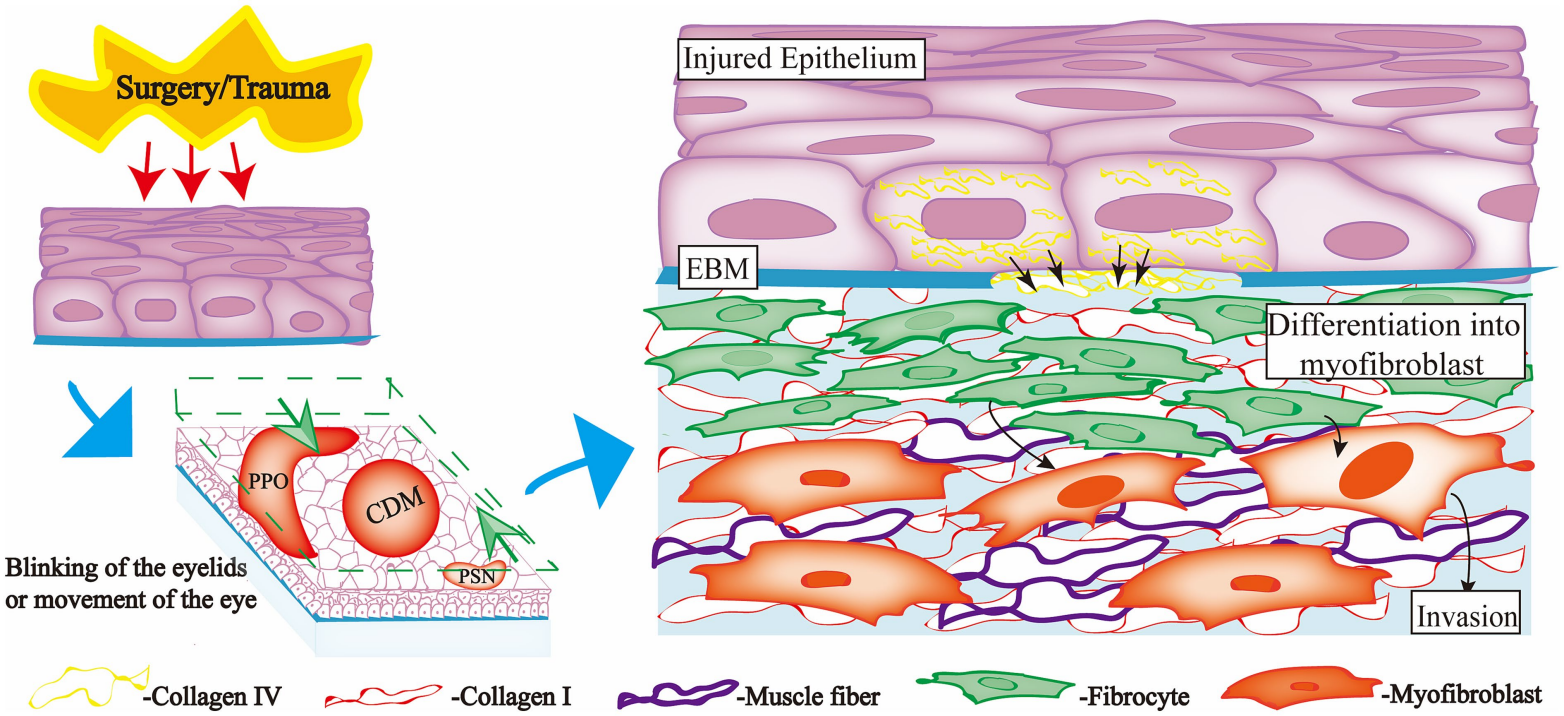
Proposed pathogenic mechanism of ACSH. According to the results of this study, the process includes surgery/trauma-induced epithelial injury, disruption of the epithelial basement membrane with altered Collagen IV distribution, and the presence of vimentin-positive fibrocytes, α-SMA-positive myofibroblasts, and Collagen I deposits. It is further inferred from the literature that fibrocyte-to-myofibroblast differentiation and mechanical stimulation from eyelid blinking contribute to lesion formation and the development of the three ACSH phenotypes (PPO, PSN, CDM).

Potential predisposing factors were explored based on AS-OCT and immunofluorescence findings. Corneal epithelial injury is recognized as a primary trigger for various degenerative corneal disorders (8, 9), and discontinuity of the epithelial basement membrane (EBM) may induce subepithelial fibrosis and myofibroblast activation (10, 11). In our cohort, type IV collagen exhibited a discontinuous linear distribution beneath the epithelium and was expressed in the cytoplasm of CK14-positive basal cells, suggesting ongoing tissue remodeling after EBM disruption. Predisposing events differed among ACSH subtypes: central diffuse lesions were more often associated with trauma, while paracentral patchy or peripheral sectorial nodules commonly occurred following corneal surgery. Mechanical friction from eyelid movement may also contribute to lesion progression. Fibrocytes and myofibroblasts appear to play key roles in remodeling, as supported by the expression of vimentin, *α*-smooth muscle actin, and type I collagen (5, 10–14). The expression of vimentin, α-smooth muscle actin, and type I collagen detected in the present study is consistent with these previous findings (5, 13, 14). These findings support the hypothesis that primary corneal injury, whether traumatic or surgical, can trigger ACSH through excessive wound repair and disordered extracellular matrix deposition, while preserving Bowman’s layer and stromal integrity (10–14). The proposed pathogenic mechanisms are summarized in Figure 9.

It is important to acknowledge that ACSH is rare and underrecognized. This study was designed as an exploratory case series to describe the clinical, imaging, and histopathological spectrum of ACSH rather than to provide definitive epidemiological or prognostic conclusions. The small sample size limits both the statistical power and the generalizability of these findings. Validation in larger cohorts with extended follow-up will be necessary. Nevertheless, our results provide a detailed characterization of ACSH phenotypes and clinicopathological features, highlight the utility of AS-OCT for objective morphological assessment, and offer guidance for early recognition and timely intervention, which may reduce Bowman’s layer and stromal damage and minimize the need for unnecessary penetrating keratoplasty.

## 5 Conclusion

This case series describes three phenotypic patterns of ACSH—paracentral patchy opacity (PPO), peripheral sectorial nodules (PSN), and central diffuse mass (CDM)—and demonstrates the value of AS-OCT in characterizing superficial corneal lesions. In treated cases, superficial keratectomy restored corneal clarity during the available follow-up period. Larger studies with standardized functional outcomes and longer follow-up are needed to validate this classification and to assess long-term recurrence risk.

## Data Availability

The original contributions presented in the study are included in the article/supplementary material, further inquiries can be directed to the corresponding author.
